# Migration phenology and breeding success are predicted by methylation of a photoperiodic gene in the barn swallow

**DOI:** 10.1038/srep45412

**Published:** 2017-03-31

**Authors:** Nicola Saino, Roberto Ambrosini, Benedetta Albetti, Manuela Caprioli, Barbara De Giorgio, Emanuele Gatti, Felix Liechti, Marco Parolini, Andrea Romano, Maria Romano, Chiara Scandolara, Luca Gianfranceschi, Valentina Bollati, Diego Rubolini

**Affiliations:** 1 Department of Biosciences, University of Milan, via Celoria 26, I-20133 Milan, Italy; 2 Department of Earth and Environmental Sciences (DISAT), University of Milano Bicocca, Piazza della Scienza, 1, I-20126 Milan, Italy; 3 EPIGET - Epidemiology, Epigenetics and Toxicology Lab - Department of Clinical Sciences and Community Health, University of Milan, via San Barnaba 8, I-20122 Milan, Italy; 4 Swiss Ornithological Insititute, Seerose 1, CH-6204, Sempach, Switzerland

## Abstract

Individuals often considerably differ in the timing of their life-cycle events, with major consequences for individual fitness, and, ultimately, for population dynamics. Phenological variation can arise from genetic effects but also from epigenetic modifications in DNA expression and translation. Here, we tested if CpG methylation at the poly-Q and 5′-UTR loci of the photoperiodic *Clock* gene predicted migration and breeding phenology of long-distance migratory barn swallows (*Hirundo rustica*) that were tracked year-round using light-level geolocators. Increasing methylation at *Clock* poly-Q was associated with earlier spring departure from the African wintering area, arrival date at the European breeding site, and breeding date. Higher methylation levels also predicted increased breeding success. Thus, we showed for the first time in any species that CpG methylation at a candidate gene may affect phenology and breeding performance. Methylation at *Clock* may be a candidate mechanism mediating phenological responses of migratory birds to ongoing climate change.

Organisms fine-tune the timing of their life-cycle events (i.e. their phenology) according to circannual variation in ecological conditions^1^. Temporal orchestration of life-cycle events such as budding and flowering in plants, and breeding and migration in animals, is a major target of natural selection, which sets a fitness premium on temporal matching of these activities with the ecological conditions when they are best performed^1^,^2^,^3^. Variation in the time schedule of major life-history events among individuals, however, can be large, and our understanding of the mechanisms that generate such variation is lagging behind the appreciation of its pervasive occurrence. In particular, no studies to date have addressed the role that DNA epigenetic modifications may have in shaping the phenology of animals. However, recent studies have started to unveil the role of epigenetic processes in causing variation in behavioural traits in the wild, like novelty seeking^4^, personality^5^, or migration propensity^6^.

Perception of day length (photoperiod) variation is a core mechanism that dictates the timing of seasonal behavioural and physiological changes of several organisms^7^,^8^. ‘Circadian clock’ systems sensing seasonal changes in light/dark cycles are conserved in organisms as taxonomically diverse as plants and vertebrates^9^,^10^. *Clock* genes, in particular, code for elements of a transcriptional-translational feedback loop (‘core circadian oscillator’; CCO)^9^. Within CCO, *Clock* and *Bmal1* products heterodimerize to produce a transcription factor, which acts both as a positive driver of the molecular oscillations and as an ‘output’ signal from the circadian oscillator^11^. The central pacemakers in the avian pineal gland, retinae and SCN dynamically interact to maintain stable phase relationships and then influence downstream rhythms through entrainment of peripheral oscillators in the brain controlling behavior^12^. An important role in controlling circadian rhythms is played by the *Clock* carboxyl-terminal polyglutamine repeat (poly-Q) domain, which serves as a transcriptional trans-activation domain^13^ and appears to be highly evolutionarily conserved^14^.

*Clock* polymorphism predicts among-individual variation in breeding, migration and molt time schedules in some species^15^,^16^,^17^,^18^, although evidence is inconsistent across species and phenological traits^18^,^19^,^20^,^21^. In the focal species of the present study, the barn swallow (*Hirundo rustica*), *Clock* exhibits low polymorphism at the poly-Q region^16^,^22^. Albeit low, however, polymorphism at poly-Q predicts timing of spring migration and breeding, and of plumage molt in the sub-Saharan wintering quarters, with the individuals carrying alleles with larger *Clock* poly-Q region showing delayed phenology^16^,^22^,^23^.

Strong genetic determinism of phenological traits may evolve because of the advantages that strict endogenous control of circannual rhythmicity can afford in terms of appropriate timing of life history events^24^,^25^. ‘Hardwired’ mechanisms of phenological control relying on photoperiodic information may be favoured because seasonal changes of environmental conditions are predictably associated with change in photoperiod. Yet, genetic variation at the few candidate phenological genes that have been investigated so far, like *Clock* and other photoperiodic genes, can be small even in populations showing large phenological variation^15^,^16^, rather suggesting low environmental canalization of phenological traits. In fact, environmental conditions can be a major source of phenological variation via their effects on individual general state, because major life-history events like reproduction and migration are energy and time demanding, and individual physical conditions can thus constrain individual phenological decisions^26^.

Epigenetic processes can reconcile small genetic variation in phenological genes with often large among-individuals variation in phenology, and thus also with the observed phenological responses of populations to climate change^27^. Environmental experience can orchestrate the way in which genetic information is expressed and translated, and epigenetic DNA alterations can thus mediate the effects of environmental conditions on phenotypic expression^28^. Methylation at CpG dinucleotides located in CpG islands, for example, has been shown to correlate with a stable shutdown of the associated promoter, and consequently a block of gene transcription^29^.

Here, for the first time we analyzed individual variation in major phenological events (i.e. start and end dates of spring migration, and breeding date) in relation to methylation at two regions (poly-Q and 5′-UTR) of the photoperiodic *Clock* gene^30^. We focused on long-distance migratory barn swallows that were equipped with light-level geolocators to record the timing of spring migration from sub-Saharan Africa to the breeding sites in southern Europe. We expected methylation at both loci to result in earlier spring migration and breeding phenology, because methylation is usually associated with a reduced transcription and thus an expected weaker activation signal from the CLOCK/BMAL1 transcription^31^,^32^,^33^,^34^,^35^. Importantly, epigenetic components of phenotypic variation may not be independent of underlying genetic variation. In the analyses, we therefore controlled for *Clock* genotype, expressed as number of the poly-Q repeats at the *Clock* region, because genetic polymorphism at poly-Q has been shown to predict phenological variation in barn swallows^16^,^23^. The methodological and technical information on genotyping of the *Clock* poly-Q region and on the relationships between genotype and migration phenology have been reported in a previous study^23^.

In addition, we analyzed the consequences of methylation at *Clock* for seasonal breeding success. The link between methylation and breeding success could be an indirect one, being mediated by the cascading ‘carry-over’ effects^36^ of timing of migration on timing of breeding and thus number of breeding events per season. Under this scenario larger methylation was expected to result in larger seasonal reproductive success because larger methylation was predicted to advance migration and breeding dates and thus to increase the seasonal number of broods. In an attempt to discern between pattern and process we therefore partitioned the direct and indirect (mediated by phenology) contributions of methylation to breeding success by means of path analysis.

## Results

Methylation at both loci did not significantly differ between males and females (poly-Q: males: 74.82% (0.99, n = 65), females: 77.11% (1.11, n = 29); t_92_ = 1.37, P = 0.173; 5′-UTR: males: 80.95% (0.43, n = 66), females: 81.27% (0.79, n = 28), t_92_ = 0.39, P = 0.696).

Methylation levels at poly-Q recorded in two consecutive years (i.e. the years when the geolocator was deployed or, respectively, when it was recovered) were positively correlated (r = 0.470, n = 25, P = 0.018), whereas there was no significant correlation between years in methylation at the 5′-UTR (r = 0.141, n = 23, P = 0.521). Hence, methylation levels at poly-Q at one life stage reflect methylation also at other life stages.

### Methylation and spring phenology

**Figure 1:**
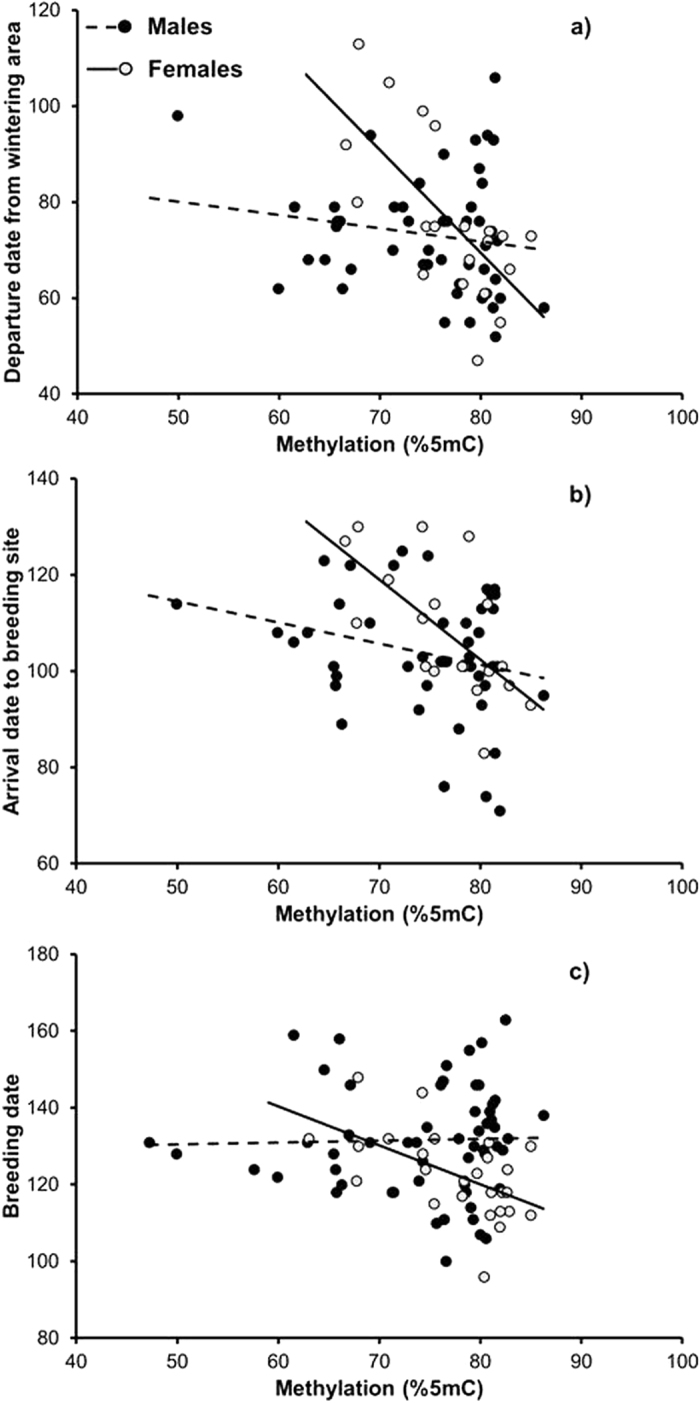
Phenological variation in departure date from the wintering area (**a**), arrival date at the breeding site (**b**), and breeding date (**c**) of male (full symbols) and female (open symbols) barn swallows in relation to methylation at the poly-Q locus. Linear regression lines for males (dashed) and females (continuous) are shown. Dates are expressed as Julian day (1 = 1 January). See Table 1 for sample sizes.

Departure date from the wintering area was significantly earlier in females but not in males with higher levels of poly-Q methylation (Table 1 and Fig. 1a). Arrival date to the breeding site was significantly earlier in both males and females with increased poly-Q methylation (Table 1 and Fig. 1b). In addition, breeding date was significantly earlier in females but not in males with relatively high poly-Q methylation (Table 1 and Fig. 1c). For all phenological variables, the relationship with poly-Q methylation was significantly steeper and negative among females, as implied by the significant sex by methylation interaction effect (Table 1 and Fig. 1). The proportion of variance in phenology of females that was explained by methylation was as large as 50.0% for departure date from the wintering area, 42.3% for arrival date to the breeding site and 29.9% for breeding date. The corresponding values for males were considerably smaller: 2.8%, 6.8% and 0.10%, respectively. Thus, the size of the effect of methylation on migration and breeding phenology was larger in females as compared to males.

In barn swallows, phenological traits including timing of spring migration and breeding, and molt schedules during wintering are predicted by genetic polymorphism at the poly-Q Clock regions^16^,^22^,^23^. We therefore re-ran the models reported in Table 1 for poly-Q while also including the effect of mean within-individual number of poly-Q repeats or, respectively, the number of poly-Q repeats of the ‘longer’ allele as covariates (see also refs 16,18). The results of these models were qualitatively consistent with those in Table 1, meaning that all the significant interaction effects and also the within-sex relationships in Table 1 remained statistically significant (details not shown). Hence, the effect of methylation at *Clock* poly-Q on phenology were independent of the underlying individual genetic constitution at *Clock* poly-Q.

Methylation at 5′-UTR, however, did not predict any of the phenological variables, either *per se* or in combination with the effect of sex (Table 1).

### Methylation and seasonal breeding success

**Figure 2:**
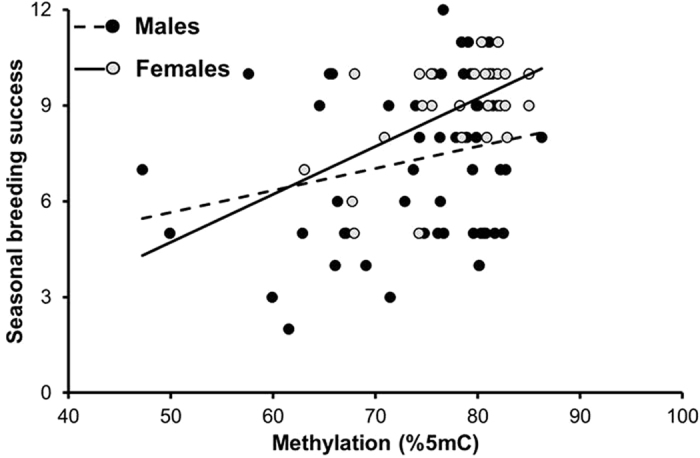
Seasonal breeding success of male (full symbols) and female (open symbols) barn swallows in relation to methylation at the poly-Q locus. Linear regression lines for males (dashed) and females (continuous) are shown. See Table 2 for sample sizes.

Methylation at poly-Q positively predicted seasonal breeding success (total number of eggs), with no statistically significant evidence for sex-dependent effects (Table 2). This result implies that the slopes of the relationships between breeding success and methylation did not differ between the sexes (Table 2 and Fig. 2). However, the correlation coefficient between breeding success and methylation at poly-Q was statistically significant for females (r = 0.552, R^2^ = 0.305; P = 0.003, n = 26) but not for males (r = 0.226, R^2^ = 0.051, P = 0.082, n = 60). Methylation at 5′UTR did not predict breeding success (Table 2).

### Path analysis of the effect of methylation on breeding success

**Figure 3:**
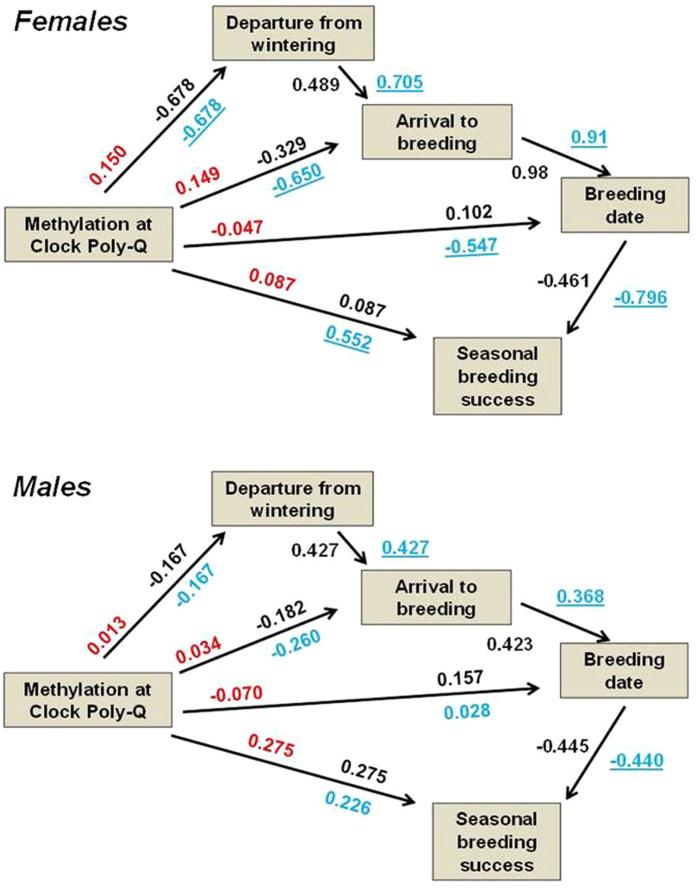
Path diagram of the direct or the indirect effect of methylation at the poly-Q mediated by spring migration phenological variables and breeding date on breeding success for females and males. Black numbers: path coefficients (i.e. the standard partial regression coefficient that estimates the strength of the relationship between each putative causative variable and the corresponding effect). Red numbers: products of the chains of path coefficients along all the paths connecting methylation at *Clock* poly-Q and seasonal breeding success; the sum of these products is used to compute the compound correlation between methylation at *Clock* poly-Q and seasonal breeding success (see Results). Blue numbers: simple correlation coefficients between pairs of variables. Underlining of correlation coefficients indicates statistical significance (P < 0.05). See Tables 1 and 2 for sample sizes.

Phenological events occur in ordered sequences and each of them is therefore potentially influenced by timing of the preceding events^36^. Such carry-over effects can therefore have cascading effects through the annual cycle, generating correlations between dates of phenological events as well as correlations with fitness traits (see ref. 37). In female barn swallows, departure date from the wintering area positively predicted arrival date at the breeding site, which in turn positively predicted breeding date (Fig. 3). Moreover, females that started breeding early had larger seasonal reproductive success (Fig. 3). In males, there were also positive relationships between the dates of consecutive phenological stages, and a negative relationship between breeding date and seasonal breeding success, although the size of the effect was smaller than in females (Fig. 3). Hence, any association between methylation and breeding success could result either from a direct effect or through an indirect effect mediated by phenology. Path analysis suggested that in females methylation at poly-Q affected breeding success mostly indirectly, through the relationships with date of departure from the wintering area and date of arrival to the breeding site, whereas the indirect effect via breeding date and the direct effect on breeding success were smaller (Fig. 3). In contrast, in males most of the effect of methylation was a direct one (Fig. 3). The overall compound path coefficients for the effect of methylation at poly-Q on breeding success was somewhat larger for females (0.339) as compared to males (0.252).

## Discussion

Variation in ecologically important traits can be generated by epigenetic modifications of the expression and function of genes even when genetic variation at those traits is small. Epigenetic processes may therefore be pivotal to our understanding of how organisms respond to environmental variation via phenotypic plasticity^27^. Ecological and evolutionary studies have long sought for the proximate mechanisms that control individual variation in the timing of major life-history events, because such variation has consequences for individual fitness and for the dynamics and evolution of populations. Here, we showed, for the first time in any species, that epigenetic regulation via methylation of a *Clock* poly-Q region can have a major impact in regulating timing of migration and breeding. Methylation at *Clock* poly-Q explained a very large fraction (>30%) of the variance in spring migration phenology, arrival dates at the breeding site and thus in seasonal breeding success, particularly in females. In males, the effects of methylation on spring migration phenology were considerably weaker than in females, and the consequences of methylation for seasonal breeding success seemed to be independent of timing of spring migration and breeding date. The sign of the relationship between methylation levels and dates of phenological events, with larger methylation levels being associated with earlier phenology, was consistent with the expectation since hypermethylation is expected to reduce the overall quantity of the CLOCK/BMAL1 transcription factor, thus reducing the transcriptional activation of the clock genes. Such reduction may possibly mimic the condition of small length of the poly-Q stretch, which is associated with earlier phenology of breeding, migration and molt^16^,^22^,^23^, although with a different mechanism, as the poly-Q region is directly involved in the transcriptional trans-activation of the CLOCK/BMAL1 regulated genes. The effect of methylation on phenology that we documented here, however, was independent of genetic polymorphism at *Clock* poly-Q, as demonstrated by analyses where we controlled statistically for the effect of length of the *Clock* poly-Q repeat. It should be noted that in the present study we measured *Clock* methylation in peripheral blood because for ethical reasons we could not sample other tissues (e.g. brain). While the present results should be considered under this caveat, methylation differences measured in blood have been suggested could have potential for being a good proxy of the changes occurring in other tissues, in particular at a central level^38^.

As a photoperiodic gene, *Clock* has a major role in controlling endogenous circadian rhythmicity^12^,^39^. However, by interacting with exogenous cues, circadian genes like *Clock* can be entrained by local environmental factors thereby participating in the regulation of seasonality in behavioural and physiological traits^10^, and genetic variation at *Clock* genotype has been shown to predict phenological variation in breeding, migration or plumage molt in several animals from diverse taxa^15^,^18^,^22^,^23^,^40^. A causal link between methylation at *Clock* and phenological variation at photoperiod-dependent activities, like migration in birds, is suggested by the observation that DNA methylation can affect circadian rhythmicity^41^,^42^. In addition, methylation at genes of the circadian rhythm system, including those that influence responsiveness to light can also impact on discrete behavioural traits like migratoriness (i.e. being migrant or not) and on related morphological and physiological syndromes, as observed for smoltification in rainbow trouts (*Onchorhyncus mykiss*)^6^. Hence, methylation of genes of the circadian system may not only influence timing of migration and of breeding, as suggested by the present study, but also cause discontinuous variation in migratoriness and related phenotypic syndromes.

The association between migration phenology and methylation at *Clock* poly-Q in our study was apparent only among females, while methylation levels did not differ between the sexes. This result may suggest differentially larger phenological control by methylation at *Clock* in females. Thus, in females methylation at *Clock* could constrain early departure from the wintering grounds, thereby generating carry-over effects on timing of subsequent migration and arrival to the breeding site and, ultimately, affecting seasonal breeding success^37^. From a different, functional perspective, however, variation in methylation could be a mechanism enforcing *adaptive* variation in timing of start of migration. Decisions on timing of migration may have to be tuned according to the ecological conditions experienced during wintering and their effects on individual physiological state or, for example, the progress of the single annual molt that barn swallows normally complete during wintering^43^. Ecological conditions during wintering may directly affect methylation at *Clock* thereby allowing barn swallows to tune their migration phenology according to their physiological state. Hence, variation in methylation may serve as an adaptive, condition-dependent epigenetic control mechanism of individual timing of migration. Under the hypothesis that variation in methylation depends on ecological conditions during wintering, an ultimate, functional interpretation of the differentially larger effect of methylation at *Clock* poly-Q on phenology of females compared to males is that performance during spring migration and subsequent breeding performance of females is more dependent on the carry-over effects of condition during wintering as compared to males, thereby requiring stronger epigenetic control^37^ (see also Fig. 3).

The proximate mechanisms that cause variation in methylation levels between barn swallows and sex-dependence of the effects of methylation on phenology are open to speculation. Methylation of DNA has been shown to be affected by a host of environmental factors, ranging from nutrition to psychological stress and exposure to environmental pollution^30^,^44^, but our knowledge of which factors are effective on animals in the wild is nihil. Yet, ample variation in nutritional conditions and exposure to diverse form of environmental stress may exist among individual barn swallows during the wintering period, making any potential effects of such exogenous factors on methylation levels most likely to operate.

Intriguingly, in both sexes seasonal breeding success was predicted by methylation levels at *Clock* poly-Q, but path analysis suggested partly different causative links between methylation, spring migration and breeding phenology, and seasonal breeding success in either sex. In females, positive carry-over effects of methylation levels on breeding success were largely mediated by timing of spring migration and arrival to the breeding site, which depends on methylation and strongly predicts seasonal reproductive success mostly via an effect on number of breeding events per season. Indeed, early breeding translates into larger number of broods and thus reproductive output per breeding season^37^. In males, the relationship between methylation and seasonal breeding success was weaker compared to females, with a 6-fold difference in the variance in seasonal reproductive success being explained by methylation in either sex. Contrary to females, however, according to path analysis in males this relationship was mostly a direct one, being largely independent of any relationship between timing of spring migration and methylation. Hence, for the first time our study shows that methylation at *Clock* poly-Q have consistent implications for seasonal reproductive in both sexes, but the mechanisms underpinning this relationship are different in either sex.

Epigenetic processes may boost the evolutionary potential of populations when faced with environmental challenges^27^. The present results therefore have a bearing for the interpretation of the consequences of ongoing environmental change, including rapid climate change, on animal populations. Long-term studies of widely diverse organisms, like plants, insects and vertebrates have demonstrated that average phenological traits are rapidly changing within populations^45^,^46^,^47^. These temporal phenological trends have been largely interpreted as a response to climate change, and specifically to increase in temperatures at temperate and high boreal latitudes^48^. The nature of such responses, however, is far from being ascertained. Micro-evolutionary change has often been invoked^48^,^49^, although change in the genetic composition of populations has not been demonstrated, and has only been inferred indirectly. In addition, some estimates of the rate of evolutionary change in bird phenology would be inconsistent with a ‘sustainable evolution’ scenario, with selection for phenological change being not compatible with population persistence, according to theoretical models^50^,^51^. Our results suggest that temporal phenological trends may arise as a consequence of epigenetic changes in the orchestration of photoperiodic genes that control phenology. Future ecological studies may therefore seek for the ultimate causes and mechanisms that can translate environmental experience into variation of epigenetic DNA modifications. In addition, they should strive to assess whether epigenetic processes have the potential to cause trans-generational effects by generating heritable variation phenology and other fitness-related traits. Finally, in the context of the study of climate change effects, they should search for empirical evidence that methylation at phenological genes can have consequences for long-term temporal variation in phenology of populations.

## Methods

### Model species

The barn swallow is a small (ca. 20 g), long-distance migratory passerine bird^52^,^53^. Western Palearctic breeding populations almost exclusively winter south of the Sahara Desert, with some migratory connectivity^54^. Autumn migration occurs in August-October whereas pre-breeding, spring migration occurs in February-May^53^,^55^. Barn swallows are aerially insectivorous and socially monogamous, and mostly breed semi-colonially in rural buildings. Breeding pairs have up to 3 clutches (1–7 eggs each) per breeding season.

### Field procedures

We studied barn swallows at a total of 29 breeding colonies ( = farms) in southern Switzerland (46°09′N, 8°55′E) and northern Italy (two neighbouring areas: 45°33′N, 8°44′E; 45°19′N, 9°40′E)^55^,^56^, during spring-summer 2010–2012. In all study years, we captured the adults at the breeding colonies and individually marked them with numbered metal and color rings. We identified the composition of the breeding pairs by direct observation of individual markings at the nest, and monitored the breeding activities to record breeding date ( = Julian date of laying of the first egg in the clutch), and clutch size (number of eggs at clutch completion). Laying date of the first clutch was chosen as the best proxy for seasonal timing of start of breeding (‘breeding date’). Total number of eggs laid by a female or, for males, by their social female mate was computed as the sum of the size of the clutches over the entire breeding season. Seasonal total number of eggs was chosen as the best proxy for seasonal fecundity, because nestling mortality is very low in our study areas (<5% of the nestlings in broods that produce any nestlings) and hatching failure rate is also low (7–10%) (N.S. and C.S. personal observation on the focal study population; see also ref. 52). Hence, clutch size strongly determines seasonal breeding success^37^.

Full details of the geolocator deployment procedures have been reported in related studies^55^,^56^. Briefly, in summer 2010 and 2011, light-level geolocators (SOI-GDL2.10; SOI-GDL2.11) were deployed using a leg-loop harness^55^,^56^. Geolocators were then removed upon first capture of the individuals returning from migration in the year (2011 or 2012) following that of geolocator deployment. Thus, phenological information refers to wintering and breeding seasons preceding geolocator recovery. Because geolocators were deployed on adult birds in spring-summer, all the phenological information refers to individuals older than 1 year, implying that age effects, which are known to affect phenology during the first year after hatching^52^ should have not affected the results.

### Phenological variables

Light data recorded by light-level geolocators were used to obtain information on phenological variables as reported in a previous study^55^. For the purposes of the present study we estimated the departure date from the wintering area, as the Julian date of the end of the (last) ‘stationary period’ in the wintering range south of the Sahara Desert; and the arrival date to the breeding site, as the Julian date of arrival to the breeding colony in the year when the geolocator was recovered.

### Analysis of methylation at Clock poly-Q and 5′-UTR

Upon capture we collected a blood sample from all individuals by puncturing the brachial vein for methylation analysis in blood cells. Genomic DNA from clotted whole blood, previously rehydrated with an aqueous solution containing 20% sodium dodecyl sulphate (SDS) and 1 M Tris (pH 8.0), was extracted using the QIAamp DNA Mini kit 250 (#51306). The analysis of methylation was conducted on two CG loci of the *Clock* gene: one close to the ATG start-codon in the 5′-UTR and the second in the last exon of the gene where the poly-Q region is present (hereafter ‘poly-Q’ for brevity). As the complete genomic sequence of *Clock* in the barn swallow is not available, the two loci were identified as previously described in details^30^.

DNA methylation was quantified by bisulfite-PCR and pyrosequencing^57^. Bisulfite treatment deaminates unmethylated cytosine to produce uracil in DNA, while methylated cytosines are protected from the conversion to uracil, allowing a quantification of DNA methylation^58^. Briefly, the samples were bisulfite-treated using EZ DNA Methylation-Gold™ Kit (Zymo Research, Orange, CA, USA) and PCR-amplified using primers and conditions illustrated in Romano *et al*.^30^. The degree of methylation was expressed as percentage of 5-methyl-cytosines (%5mC) over the sum of methylated and unmethylated cytosines. Every sample was tested in duplicate for each marker to confirm reproducibility of the degree of *Clock* methylation. Repeatability estimates (intra-class correlation coefficient) were 0.481 (*χ*^2^_1_ = 18.26, P < 0.001) for *Clock* poly-Q exon and 0.413 (*χ*^2^_1_ = 10.71, P < 0.001) for 5′-UTR.

### Statistical analyses

We used Gaussian linear mixed models to analyze variation in phenological variables and seasonal number of eggs in relation to sex (fixed effect) and percentage methylation at either focal loci (continuous covariate). In the models, the interaction term between percentage methylation and sex was initially included, and then excluded when statistically non-significant. In all models, year (2 levels) and area (3 levels) were included as random factors. The effect of year or area were tested by likelihood ratio tests comparing the model including or, respectively, excluding each random effect at a time.

Path analysis^59^ was used to describe the direct and the indirect effects, as mediated by phenological variables, of methylation on breeding success. In reporting on the results of path analysis we show: 1) the path coefficients (i.e. the standard partial regression coefficient that estimates the strength of the relationship between each putative causative variable and the corresponding effect; see numbers in black in Fig. 3); 2) the products of the chains of path coefficients along all the paths connecting methylation at *Clock* poly-Q and seasonal breeding success (numbers in red in Fig. 3). The sum of these products was used to compute the compound correlation between methylation at *Clock* poly-Q and seasonal breeding success; and 3) the simple correlation coefficients between pairs of variables (numbers in blue in Fig. 3).

Parameter estimates are reported with their associated standard error.

Sample sizes for the different analyses (see Tables 1 and 2) slightly differ because phenological/breeding success information was not available for some individuals, as did the information for methylation.

All methods were carried out in accordance with the relevant guidelines and regulations. All experimental protocols were approved by the Istituto Superiore per la Protezione dell’Ambiente, and by Regione Lombardia, Regione Piemonte and by the Swiss Federal Office for Environment.

### Data Availability

The datasets generated during the current study are available from the corresponding author on reasonable request.

## Additional Information

**How to cite this article:** Saino, N. *et al*. Migration phenology and breeding success are predicted by methylation of a photoperiodic gene in the barn swallow. *Sci. Rep.* **7**, 45412; doi: 10.1038/srep45412 (2017).

**Publisher's note:** Springer Nature remains neutral with regard to jurisdictional claims in published maps and institutional affiliations.

## Figures and Tables

**Table 1:** Linear mixed models of phenological variables in relation to sex and methylation at *Clock* poly-Q or 5′-UTR.

	poly-Q					5′-UTR				
	χ^2^_1_	F	df	P	Coefficient (SE)	χ^2^_1_	F	df	P	Coefficient (SE)
**Departure date from wintering area**										
Year	0.00					0.00^a^				
Area	0.00					0.00^a^				
Sex		10.79	1,62	0.002			0.81^a^	1,61	0.371	
Methylation		17.43	1,62	<0.001			0.13^a^	1,61	0.717	
Sex × Methylation		10.20	1,62	0.002			1.54	1,60	0.219	
Males					−0.29 (0.24)					0.57 (0.56)
Females					−2.15 (0.53)***					−0.58 (0.75)
**Arrival date to breeding site**										
Year	9.30**					5.40^a^*				
Area	3.00					3.70^a^				
Sex		4.38	1,57	0.041			2.16^a^	1,57	0.147	
Methylation		17.64	1,57	<0.001			0.60^a^	1,57	0.440	
Sex × Methylation		3.74	1,57	0.058			0.00	1,56	0.997	
Males					−0.59 (0.22)**					−0.31 (0.52)
Females					−1.61 (0.50)**					−0.31 (0.67)
**Breeding date**										
Year	7.70**					7.70^a^**				
Area	4.30*					4.30^a^*				
Sex		3.67	1,77	0.059			6.39^a^	1,78	0.014	
Methylation		4.97	1,77	0.029			2.65^a^	1,78	0.108	
Sex × Methylation		4.60	1,77	0.035			1.45	1,77	0.232	
Males					−0.02 (0.19)					−0.95 (0.47)
Females					−0.97 (0.40)*					0.14 (0.77)

*0.05 > P > 0.01; **0.01 ≥ P ≥ 0.001; ***P < 0.001. ^a^Estimated from a model excluding the interaction term. χ^2^_1_: likelihood ratio test for the random effect of year or area. Methylation (percentage of 5-methyl-cytosine) is included as a covariate. Year and area are included as random factors; sex as a fixed-effect factor. Sample sizes for the analyses of poly-Q were as follows (males, females): departure date from wintering area: 49, 20; arrival date to breeding site: 45, 19; breeding date: 58, 26. The corresponding values for the analyses of 5′-UTR were: 48, 19; 45, 18; 59, 25; 61, 25.

**Table 2:** Linear mixed models of seasonal breeding success in relation to sex and methylation at *Clock* poly-Q or 5′-UTR.

	poly-Q					5′-UTR				
	χ^2^_1_	F	df	P	Coefficient (SE)	χ^2^_1_	F	df	P	Coefficient (SE)
Seasonal fecundity										
Year	0.00^a^					0.00^a^				
Area	0.50^a^					0.70^a^				
Sex		5.86^a^	1,80	0.018			6.92^a^	1,80	0.010	
Methylation		7.01^a^	1,80	0.010			0.00^a^	1,80	0.969	
Sex × Methylation		1.02	1,79	0.316			0.01	1,79	0.924	
Males					0.07 (0.03)					−0.00 (0.09)
Females					0.15 (0.07)*					0.01 (0.14)

*0.05 > P > 0.01; ^a^estimated from a model excluding the interaction term. χ^2^_1_: likelihood ratio test for the random effect of year or area. Methylation (percentage of 5-methyl-cytosine) is included as a covariate. Year and area are included as random factors; sex as a fixed-effect factor. Sample sizes for the analyses of poly-Q were 60 males and 26 females. Sample sizes for analyses of 5′-UTR were 61 males and 25 females.
